# Surgical Diagnosis and Treatment of Primary Retroperitoneal Liposarcoma

**DOI:** 10.3389/fsurg.2021.672669

**Published:** 2021-06-04

**Authors:** Jie Chen, Ying Hang, Qi Gao, Xinyu Huang

**Affiliations:** ^1^Department of General Surgery, Shanghai Jiaotong University Affiliated Sixth People's Hospital, Shanghai, China; ^2^Department of Emergency, Renji Hospital, School of Medicine, Shanghai Jiaotong University, Shanghai, China

**Keywords:** primary retroperitoneal liposarcoma, overall survival, surgical diagnosis, disease-free survival, management

## Abstract

**Background:** Primary retroperitoneal liposarcoma (PRPLS) is the most common soft tissue sarcoma of the retroperitoneum with high recurrence rate and short overall survival (OS).

**Methods:** A retrospective review of 51 patients with PRPLS, treated between September 1, 2009 and November 30, 2020, was conducted to evaluate clinical outcomes of PRPLS resection. Patient demographics, histopathologic subtypes, overall survival (OS), progression-free survival (PFS), disease recurrence rate, and tumor stage were reviewed and analyzed. Univariate analysis was done to identify factors potentially affecting OS and PFS of PRPLS patients. Multivariate Cox proportional hazards analysis was used to evaluate the impact of various clinicopathological factors on OS and PFS of PRPLS patients.

**Results:** Fifty-one PRPLS patients (28 Males, 23 Females; mean age 56.25 years) were evaluated. There was no significant effect of age, gender, contiguous organ resection, degree of differentiation and tumor size on the OS and PFS of the patients. Univariate analysis showed that negative surgical margin and early tumor stage significantly correlated with OS and PFS (all *P* < 0.001). Multivariate analysis showed that tumor stage [hazard ratio (HR) = 1.177, *P* = 0.001] was an independent predictors of poor progression-free survival, and surgical margins [HR = 4.0674 *P* = 0.038] and tumor stage [HR = 1.167 *P* = 0.001] were identified as independent predictors of poor overall survival.

**Conclusion:** Negative surgical margin is a prognostic factor of OS, and can prolong the postoperative survival time of PRPLS patients. Tumor stage is a prognostic factor for OS and PFS, and can influence the survival of PRPLS patients. Earlier tumor stages of PRPLS are associated with significantly better outcomes.

## Introduction

Liposarcoma is the most common soft tissue sarcoma in the adult population (1). Liposarcomas can manifest anywhere in the body, usually starting from extremities flowed by retroperitoneum and inguinal lesions. Clinical characteristics of liposarcomas closely reflect their pleomorphic histology, with large-size lesions more common found in the retroperitoneum (2, 3). The diagnosis and treatment of liposarcomas are challenging due to the lack of clinical symptoms, large size and loose structure of adult retroperitoneal space. According to the World Health Organization (WHO) classification (4), there are several histological subtypes of PRPLS: myxoid/round cell LPS, pleomorphic LPS and well-differentiated and dedifferentiated LPS (WDLPS/DDLPS) that are characterized by amplification of MDM2 and CDK4 genes on chromosome 12q13-15 (5, 6). The primary treatment option for PRPLS is surgical resection, when possible (7, 8). However, local recurrence is common and occurs in 66% of the patients (9). Previous study of a large series of complete resections showed that a five-year overall survival (OS) rate of PRPLS patients was 54% (10). The main goal of the current study is to review our strategies in the management of PRPLS in a single center and to identify some related prognostic factors.

## Materials and Methods

### Patients

One hundred and twenty-three patients with retroperitoneal tumors were identified at our institute between September 1, 2009 and November 30, 2020. Of them, 72 patients with other types of liposarcomas, such as leiomyoma, schwannoma, lymphoma, paraganglioma, angiomyolipoma and leiomyosarcoma were excluded. The remaining 51 patients were included in the current study, and their demographics, histopathologic subtypes, disease recurrence rate, tumor stage, OS and PFS were retrospectively reviewed and recorded. The study was approved by the Institutional Review Board and ethics committee.

### Assessed Parameters

The following parameters were assessed: age at diagnosis, sex, symptoms at presentation, histopathologic subtypes, surgical margin, tumor size, tumor stage, disease recurrence rate, OS and PFS. Tumor size was defined as the maximum dimension of the solitary mass on cross-sectional imaging, and as the sum of all maximal dimensions for more than one mass (11). Recurrence was defined as the time from the first operation to clinical recurrence confirmed by imaging. Computed tomography (CT), a diagnostic investigation of choice for PRPLS, was used to determine tumor location, size, and metastases (12, 13). Imaging examination (CT) was performed at 1 and 3 months after operation. If there was no recurrence within 1 year, imaging examination was performed after 6 or 12 months. Morbidity and mortality were analyzed by reviewing charts and clinical records of patients.

### Surgical Procedure

All the operations were performed by exploratory laparotomy. Thirty-one patients underwent combined organ resection, and the rest were simple tumor resection. Among them, 12 patients underwent combined multiple organ resection. According to the preoperative evaluation, all patients could tolerate the combined organ resection. Kidney was the most common organ resected, followed by ureter, colon, small intestine, spleen and pancreas. Combined urinary organ resection was done with the assistance of a urologist. Before the operation, patients were administered a routine oral laxative. In case of tumor invading the small intestine or colon, one-stage resection and anastomosis was performed. In case of tumor invading the body and tail of pancreas, combined pancreatectomy and splenectomy were performed. The goal of every type of surgical procedure was to remove the tumor completely.

### Statistical Analysis

Statistical analysis of the survival curve was performed using the Kaplan-Meier method. Univariate analysis and comparison of each factor of interest was done by Tarone and Ware test, a modification of the log rank test for comparing two survival distributions that is can provide a valid statistical test, even with a large fraction of censored data (14). The effect of various clinicopathological factors on OS and PFS was assessed by multivariate Cox proportional hazards analysis using a backward stepwise procedure (entry, 0.05; removal, 0.10). *P* < 0.05 was considered statistically significant.

## Results

### Clinico-Pathological Characteristics

Table 1 summarizes the patient's demographic, surgical and pathological data.

**Table 1:** Clinico-pathologic and treatment characteristics in patients with primary liposarcoma of the retroperitoneum.

**Variables**		**Mean/median/*n* (percentage %)**
Age		
	Mean (std)	56.25 (12.72)
	Median (range)	57.0 (50.0 65.0)
Gender (*n*, %)		
	Male	28 (54.9)
	Female	23 (45.1)
Stage (*n*, %)		
	I A	1 (2.0)
	I B	30 (58.8)
	III A	2 (3.9)
	III B	11 (21.6)
	IV	7 (13.7)
Histology (*n*, %)		
	Well-differentiated	25 (49.0)
	De-differentiated	22 (43.1)
	Myxoid	2 (3.9)
	Mixed-type	2 (3.9)
Tumor size (*n*, %)		
	< = 15 cm	20 (39.2)
	> 15 cm	31 (60.8)
Margins (*n*, %)		
	Positive	43 (87.8)
	Negative	6 (12.2)
Resection of contiguous organs (*n*, %)		
	Yes	31 (63.3)
	No	18 (36.7)

Fifty-one patients with PRPLS were evaluated (28 Males, 23 Females; mean age: 56.25 years). Most of the patients (47 out of 54) were over 40 years of age, and 4 patients were <40 years old. The main clinical symptoms included abdominal discomfort and abdominal distension. Tumor mass was palpable on most abdominal physical examinations. Of the detected masses, 25 cases were well-differentiated tumors (49%), 2 cases were myxoid cells (3.9%), 22 cases were dedifferentiated (43.1%), and 2 cases were mixed-type liposarcomas (3.9%). One patients' tumor was classified as stage I A (2%), 30 were stage I B (58.8%), 2 were stage III A (3.9%), 11 were stage III B (21.6%), and seven were stage IV (13.7%) tumors. Tumor staging is summarized in Table 2.

**Table 2:** Tumor stage evaluation.

**Stage (*n*, %)**	***n***	**% (tenths)**	**% (percentile)**
I A	1	2.0	1.96
I B	30	58.8	58.82
III A	2	3.9	3.92
III B	11	21.6	21.57
IV	7	13.7	13.73
Total	51	100.0	100.00

The average tumor size, defined as the maximum tumor diameter, was 19 cm (ranging from 5 to 47 cm). Thirty-one patients had tumor sizes larger than 15 cm, twenty patients had tumors smaller than 15 cm. Median follow-up time was 42 months (ranging from 1 to 156 months), with no perioperative mortality. One patient developed postoperative intestinal fistula. Forty-three cases (87.8%) had positive surgical margin. Thirty-one patients underwent contiguous organs resection, and 39 (76.5%) patients had tumor recurrence. There were no deaths within 30 days after surgery reported in this study. There were only two cases of perioperative complications among 51 patients. One patient developed small intestinal leakage and was treated conservatively for 3 weeks. One patient developed postoperative pulmonary infection, which was improved after anti-inflammatory treatment for 2 weeks. Patients had very few perioperative complications that did not affect the discharge time.

### Progression-Free Survival and Overall Survival Analysis

**Figure 1:**
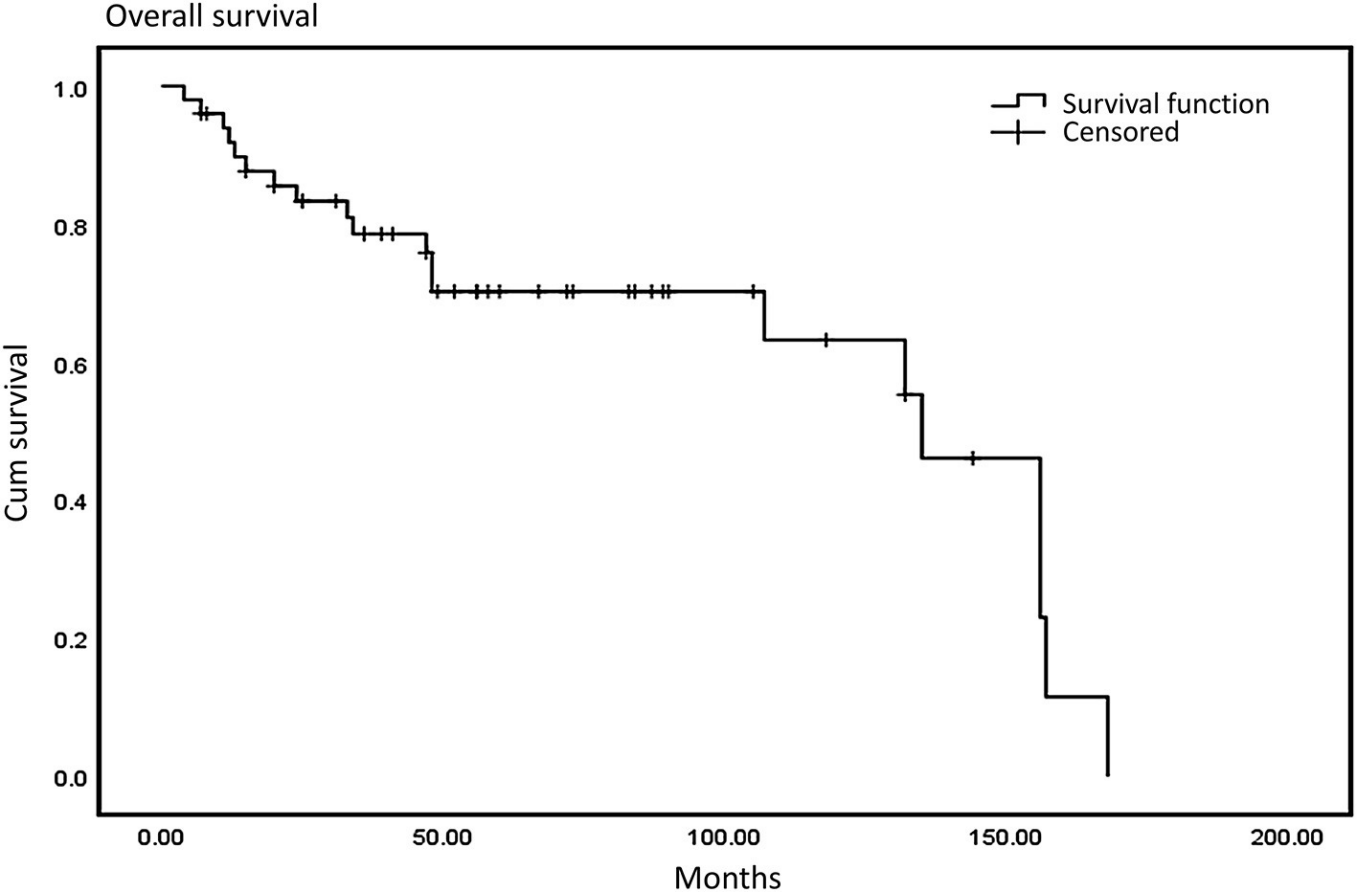
Overall survival: the median OS was 48.5 months and the 3- and 5-year OS were 62 and 38%.

**Figure 2:**
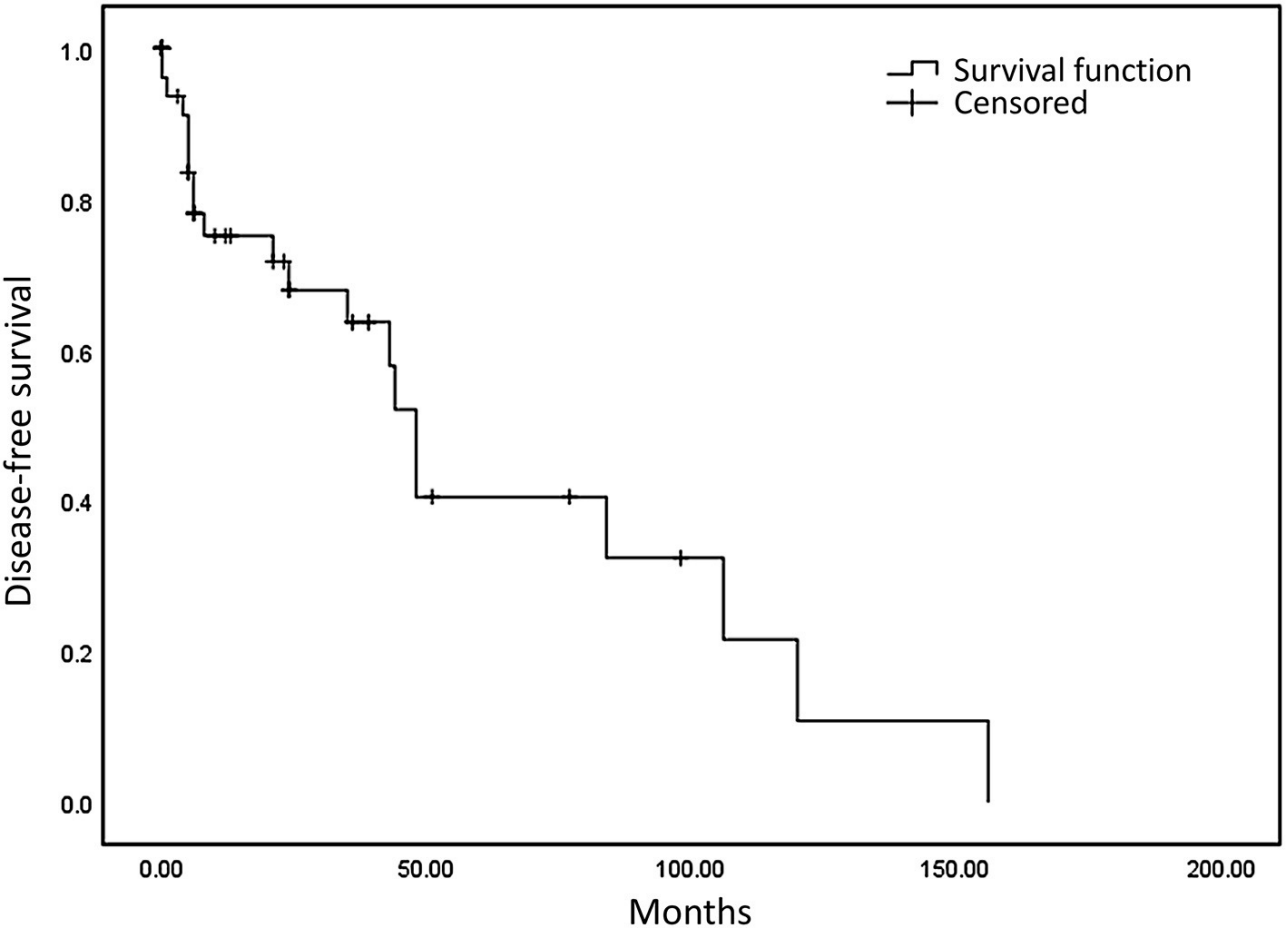
Progression-free survival: the median PFS was 23 months and the 3- and 5-year PFS were 66.2 and 42.3%.

We defined progression-free survival (PFS) as the time from the initial diagnosis to the first occurrence of disease progression, death, or death without evidence of recurrence or progression. More specifically, for patients who had an R0/R1 resection, PFS was defined as date of first recurrence; for patients who had R2 resection, PFS indicated date of progression of residual disease. We defined a negative resection margin as R0 resection and a positive or close postoperative margin (<1 mm without intact fascia) as a R1 resection. If a residual tumor was detected during an operation, R2 (palliative) was considered. Progression-free survival was more appropriate to be applied to a metastatic tumor after surgical treatment. Median PFS in our study was 23 months and median overall survival was 48.5 months. The 3-year and 5-year overall survival (OS) rates were 76.6 and 68.4%, respectively (Figure 1), and the 3-year and 5-year PFS were 66.2 and 42.3%, respectively (Figure 2).

### Univariate Analysis on Risk Factors

The univariate analysis was conducted to determine whether factors, such as gender, age, tumor size, surgical margins, degree of differentiation, tumor stage, contiguous organ resection, lesions and ascites were associated with 3- and 5-year OS/PFS rates.

#### Age and Gender

As summarized in Table 2, univariate analysis showed that female patients had a 3- and 5-year OS of 65.2 and 47.8% respectively, while male patients had a 3- and 5-year OS of 59.3 and 29.6% respectively (*P* = 0.613). The 3- and 5-year OS of patients older than 50 years old were 57.6 and 30.3%, respectively; the 3- and 5-year OS of patients younger than 50 years old were 70.6 and 52.9%, respectively (*P* = 0.428).

A 3- and 5-year PFS of female patients was 65.2 and 47.8%. Male patients had a respective 3- and 5-year PFS of 57.1 and 28.6% (*P* = 0.622). The 3- and 5-year PFS of patients older than 50 years old was 55.9 and 29.4%, respectively, whereas for the patients younger than 50 years old, the 3- and 5-year PFS was 70.6 and 52.9% (*P* = 0.704) (Table 3).

**Table 3:** Risk factors for overall survival after operation (*p*-values of the Tarone-ware test are presented).

**Risk factors**	**3-year survival rate**	**5-year survival rate**	***p*-value**
Gender			0.613
Male	59.3%	29.6%	
Female	65.2%	47.8%	
Age (>50 years)			0.428
≤ 50	70.6%	52.9%	
>50	57.6%	30.3%	
Tumor size (>15 cm)			0.174
≤ 15	59.1%	45.5%	
>15	64.3%	32.1%	
Surgical margins			<0.001
Negative	72.1%	44.2%	
Positive	0.0%	0.0%	
Degree of differentiation			0.055
Well-differentiated	68.0%	52.0%	
Not well-differentiated	56.0%	24.0%	
Tumor stage			<0.001
I A	100.0%	100.0%	
I B	80.0%	60.0%	
III A	50.0%	0.0%	
III B	45.5%	0.0%	
IV	0.0%	0.0%	
Contiguous organ resection			0.866
Yes	64.5%	32.3%	
No	61.1%	50.0%	
Lesions			0.022
Single	70.0%	50.0%	
Multifocality	56.7%	30.0%	
Ascites			0.035
Yes	44.4%	22.2%	
No	65.9%	41.5%	

#### Tumor Characteristics

The 3- and 5-year OS for patients with tumors larger than 15 cm were 64.3 and 32.1% as compared to 59.1 and 45.5% respectively in patients with tumors smaller than 15 cm (*P* = 0.174). Tumors larger than 15 cm were associated with a 3- and 5-year PFS of 64.3 and 32.1%, while patients with tumors smaller than 15 cm had 3- and 5-year PFS of 56.5 and 43.5% respectively (*P* = 0.277) (Table 3).

#### Positive Margins Are Associated With Worse OS and PFS

Both 3- and 5-year OS for patients with positive surgical margins were 0.0%, whereas in patients with negative margins 3- and 5-year OS were 72.1 and 44.2% (*P* < 0.001). Similarly, there was a 0.0% 3- and 5-year PFS in patients with positive margins, and 72.1 and 44.2% respectively in patients with negative margins (*P* < 0.001) (Table 3).

#### Earlier Tumor Stages Are Associated With Better OS and PFS

The 3- and 5-year OS for patients with well-differentiated tumors were 68.0 and 52.0% respectively, and 56.0 and 24.0% respectively for patients with tumors that were not well-differentiated (*P* = 0.055). The 3-year OS for different tumor stages was as follows: IA-100.0%, IB-80.0%, IIIA-50.0%, IIIB-45.5%, and IV-0.0%. Similarly, the 5-year OS for tumor stage IA was 100.0%, IB-60.0%, IIIA-0.0%, IIIB-0.0%, IV-0.0% respectively (*P* < 0.001). The 3-year and 5-year PFS for patients with well-differentiated tumors were 68.0%, 52.0%, and for patients with not well-differentiated tumors- 53.8 and 23.1% (*P* = 0.051). The 3-year PFS for tumor stages was as follows: IA-100.0%, IB-80.0%, IIIA-50.0%, IIIB-45.5%, and IV-0.0%; the 5-year PFS for tumor stages was IA-100.0%, IB-60.0%, IIIA-0.0%, IIIB-0.0%, and IV-0.0% (*P* < 0.001) (Table 3).

#### Association of Organ Resection With OS and PFS

The 3- and 5-year OS were 64.5 and 32.3% for patients with contiguous organ resection, and 61.1 and 50.0% respectively for patients without contiguous organ resection (*P* = 0.866) (Table 2). Kidney was the most common organ resected, followed by ureter, colon, small intestine, spleen and pancreas. Twelve patients required two or more organs resected. The 3-year and 5-year PFS for patients with contiguous organ resection were 64.5 and 32.3%, and for patients without contiguous organ resection- 61.1 and 50.0% respectively (*P* = 0.496) (Table 3).

#### Multifocality and Ascites Are Associated With Worse OS and PFS

The 3- and 5-year OS for patients with single lesions were 70.0 and 50.0%, significantly higher than that for patients with multifocality (56.7 and 30.0% respectively, *P* = 0.022). Similarly, 3- and 5-year PFS for patients with single lesions were significantly higher (70.0 and 50.0%) than that recorded for patients with multifocality (54.8 and 29.0% respectively), *P* = 0.033 (Table 3). The 3- and 5-year OS for patients with ascites were 44.4 and 22.2% respectively, while patients without ascites had 3- and 5-year OS of 65.9 and 41.5% respectively (*P* = 0.035) (Table 2). Three-year and 5-year PFS for patients with ascites were 44.4 and 22.2%, respectively, as compared to 64.3 and 40.5% in patients without ascites (*P* = 0.101) (Table 3).

#### Multivariate Analysis of the Effect of Clinicopathological Factors on Progression-Free Survival and Overall Survival After Operation

Multivariate analysis was then performed using the backward stepwise procedure to evaluate the effect of clinicopathological factors on post-surgery PFS and OS. For the first step of the analysis, all the univariately significant factors were entered in the multivariate analysis. Subsequently, the remaining factors were entered in the analysis. Final model included factors with statistically significant *P*-values (<0.05).

Results of the analysis showed that tumor stage [hazard ratio (HR) = 1.177, *P* = 0.001] was an independent predictor of poor progression-free survival (Table 4). Surgical margins [HR = 4.0674 *P* = 0.038] and tumor stage [HR = 1.167 *P* = 0.001] were also found to be independent predictors of poor overall survival (Table 5).

**Table 4:** Risk factors for progression-free survival after operation (*p*-values of the tarone-ware test are presented).

**Risk factors**	**3-year survival rate**	**5-year survival rate**	***p*-value**
Gender			0.622
Male	57.1%	28.6%	
Female	65.2%	47.8%	
Age (>50 years)			0.704
≤ 50	70.6%	52.9%	
>50	55.9%	29.4%	
Tumor size (> 15 cm)			0.277
≤ 15	56.5%	43.5%	
>15	64.3%	32.1%	
Surgical margins			<0.001
Negative	72.1%	44.2%	
Positive	0.0%	0.0%	
Degree of differentiation			0.051
Well-differentiated	68.0%	52.0%	
Not well-differentiated	53.8%	23.1%	
Tumor stage			<0.001
I A	100.0%	100.0%	
I B	80.0%	60.0%	
III A	50.0%	0.0%	
III B	45.5%	0.0%	
IV	0.0%	0.0%	
Contiguous organ resection			0.496
Yes	64.5%	32.3%	
No	61.1%	50.0%	
Lesions			0.033
Single	70.0%	50.0%	
Multifocality	54.8%	29.0%	
Ascites			0.101
Yes	44.4%	22.2%	
No	64.3%	40.5%	

**Table 5:** Multivariate analyses of the effect of clinicopathological factors.

**Risk factors**	**HR (95% CI)**	***p*-value**
**a) On progression-free survival after operation**		
Tumor stage	1.177 (1.071–1.294)	0.001
**b) On overall survival after operation**		
Surgical margins	4.074 (1.078–15.4)	0.038
Tumor stage	1.167 (1.069–1.273)	0.001

## Discussion

Primary retroperitoneal liposarcoma (PRPLS) that accounts for about 45% of primary retroperitoneal neoplasms (15), was detected in 41.8% of all liposarcoma cases (51/122) in our study. This type of tumor is equally frequent in males and females over 40–60 years of age (16), which is consistent with our results, showing that age and gender were not associated with the increased risk of PRPLS. The detection of primary retroperitoneal liposarcoma is usually late due to lack of symptoms, and therefore a tumor can reach a large size (>15 cm) by the time of diagnosis (17). There are many case reports of giant retroperitoneal liposarcomas in the literature, with the largest reported so far weighing 42 kg (18). However, our results indicate that increased tumor size (over 15 cm) was not associated with significantly worse prognosis, as indicated by OS and PFS (*p* = 0.174 and 0.277, respectively).

Almost all retroperitoneal liposarcomas (92.1% in our study) were WDLS or DDLS (19). Previous studies have shown that tumor grade is an independent predictor of the postoperative survival time in soft tissue sarcomas (3, 20, 21). Multivariate analysis demonstrated that histological grade and pathological subtype of the tumors were independent prognosis markers. Reports show that tumor differentiation is associated with better prognosis of the patient (22). In our study, we observed the correlation between the OS and PFS and greater tumor differentiation, but this difference was not statistically significant (*P* = 0.055 and 0.051 respectively). Further larger-scale studies are needed to evaluate the effect of tumor pathological subtype and histological grade on the prognosis of PRPLS patients.

Our results show the correlation between the stages of tumors and the improved prognosis. Earlier stages (IA and IB) had a 5-year OS and PFS of 100 and 60.0% respectively, while later stages resulted in extremely poor prognosis (0% for stages III and IV) (*P* < 0.001).

Interestingly, we also report a correlation between the number of lesions and the prognosis of the disease. Patients with multifocality had markedly lower OS and PFS compared with patients diagnosed with single lesions (*P* = 0.022 and 0.033 for OS and PFS, respectively), suggesting that the number of lesions may be a predictor of poor outcome.

Our study shows that the survival time of patients with ascites [a median survival time of 5 (2.85, 7.15) months] is much shorter compared to those without ascites [81 (55.78, 106.22) months]. Ascites was associated with significantly lower OS (*P* = 0.035) but did not markedly impact PFS (*P* = 0.11). Our results are in agreement with the previous reports, showing that the presence of ascites correlates with overall poor prognosis, as it may indicate peritoneal metastasis (23). Based on these results, we suggest that patients with ascites may benefit from alternative treatment approaches instead of surgery.

In our study, tumor stage in patients with primary retroperitoneal liposarcoma was an independent predictor of poor PFS, and at the same time associated with poor OS. Previous studies had reported that tumor stage was a prognostic factor for OS and PFS (24). Our results are consistent with these observations and provide further evidence that supports the importance of tumor stage in the prognosis of PRPLS. The primary treatment for PRPLS is surgical resection with a negative margin (25), and positive microscopic margin is considered a prognostic factor for PRPLS that could reduce the postoperative survival time (3, 26). In our study, multivariate analysis also confirmed that positive surgical margins is an independent predictor of OS after operation. Based on our analysis, we conclude that complete resection of tumor with histologically negative margins could provide a better chance for long-term survival of PRPLS.

CT is the most important examination method for diagnosing PRPLS. Retroperitoneal liposarcoma is usually visualized as a large encapsulated mass containing variable amounts of fatty and soft tissue components (27). Biopsy can help to clarify the pathology, but it is not generally recommended due to the probability of tumor seeding (28). Moreover, the overall diagnostic accuracy of percutaneous biopsy for liposarcoma subtypes is not high, with Ikoma et al. reporting a diagnostic accuracy of only 63% (86/137) (25).

Recurrence of retroperitoneal liposarcoma tends to happen 6 months to 2 years after resection (26), with local recurrence rates as high as 60% at five years (3, 29). Following complete resection of primary retroperitoneal liposarcoma, 50% of well-differentiated and 80% of dedifferentiated tumors recur within 5 years (30, 31). Miao et al. reported an association of folylpolyglutamate synthetase (FPGS) rs10760502 polymorphism with increased risk for primary retroperitoneal liposarcoma, and suggested that folate supplementation might be useful in decreasing tumorigenesis and preventing postoperative tumor recurrence (32).

Surgical resection with a negative margin is considered a primary treatment for PRPLS that improves local control (33). Studies show that clean microscopic margin are associated with longer postoperative survival time compared to resections with a microscopic tumor-positive margin (3, 34). In our study, positive surgical margin was associated with extremely poor prognosis (0% 3-year OS and PFS, *P* < 0.001).

Large retroperitoneal liposarcomas present unique challenges and require more aggressive surgical approach that may include multiple resections for recurrences (35). Multiple re-operations for recurrent disease may result in significant increase in long-term survival, even despite the overall higher rate of local recurrence of PRPLS compared to other sarcomas (36). Park et al. reported that when the local recurrence growth rate was >0.9 cm/month, the prognosis was poor, and patients should be considered for enrollment in clinical trials employing novel agents (37).

Complete resection of retroperitoneal sarcoma is the most important predictor of local recurrence and survival (8). Study by Mäkelä et al. (20) showed that the rate of complete resection and subsequently, postoperative survival time, is influenced by the inaccessible, deep location of retroperitoneal liposarcomas, rather than their size alone. Studies reported that the median survival of patients who underwent complete resection was 103 months, as compared to 18 months in patients undergoing incomplete resection (38). R0 resection of a large retroperitoneal liposarcoma was associated with a 85.7% five-year survival compared to 33.3% following R1 resection (39). Wang et al. suggested that extended resection that includes adjacent organs is beneficial in order to achieve radical treatment (40). Bradley et al. reported that over 50% of successful complete excisions also included adjacent organs (17). The structures most commonly resected are kidneys, ureter and large bowel. Our results were similar to the literature, with the kidney being the most common resected organ, followed by ureter, colon, small intestine, spleen and pancreas. However, the possible benefits of resection of contiguous organs still remain controversial (3, 41, 42). Although some reports are in support of en-bloc resection of uninvolved adjacent organs to improve local control (43, 44), these studies fail to show any improvement in overall survival for extended resection beyond R0. Previous studies (45, 46) have demonstrated that organ resections can reduce the local recurrence rates but do not prolong the survival time (45, 47). In agreement with these results, our study showed that resection of contiguous organs had no significant effect on the prognosis (*P* = 0.866 and 0.496 for OS and PDF, respectively). It is possible that the advanced stage of liposarcoma at the time of organ resection negatively impacted the OS.

The is no consensus regarding the efficacy of radiotherapy and chemotherapy for retroperitoneal liposarcoma. Analysis of 61 cases of retroperitoneal liposarcoma at a large institution showed that response rates to radio- and chemotherapy are low, even with doxorubicin being the first-line chemotherapy for metastatic and or unresectable disease (48). Chemotherapy and radiotherapy are ineffective for the majority of PRPLS cases, with a chemotherapy response rate of <10% (49, 50). Recent studies reported that the overall response rate of chemotherapy was 20%. Furthermore, a partial response was reached in 35% of patients in the low-grade cohort (51). Clinical response criteria assessment still remains highly controversial.

Some reports suggest that the addition of preoperative radiation therapy (RT) to wide surgical excision for RPS results in improved local control rates when compared with surgery alone (52). Radiotherapy is commonly utilized in patients with myxoid liposarcoma of the extremity (53), and several studies have shown that myxoid liposarcoma is extremely radiosensitive (54–56). Two prospective trials successfully showed favorable long term results, such as 5-year local recurrence-free (60%), disease-free (46%), and overall survival rates (61%) in patients who underwent preoperative RT for intermediate or high-grade retroperitoneal sarcoma and achieved complete (R0) or incomplete oncological clearance (R1) (30). However, a recent large open-label, randomized, phase 3 study done in 31 research institutions (EORTC-62092: STRASS) and including 266 PRPLS patients, showed that median recurrence-free survival in the radiotherapy plus surgery group was 4.5 years (95% CI 3.9 to not estimable) compared to 5.0 years (3.4 to not estimable) in the surgery alone group (hazard ratio 1.01, 95% CI 0.71–1·44; log rank *p* = 0.95), suggesting no benefit of preoperative RT. However, the local recurrence rate in the surgery group was two-fold higher than in the radiotherapy plus surgery group. This result is possibly related to the specific impact of radiotherapy on the liposarcoma cohort (57). As reported before, preoperative RT might improve the outcome in liposarcoma and in low-grade retroperitoneal sarcoma patients, as shown by subgroup analyses of abdominal recurrence-free survival by sarcoma grade and subtype (58).

As reported in a case-control propensity score-matched analysis by the National Cancer Data Base, postoperative RT increased median survival (89 months) as compared to that in no RT group (64 months. Moreover, postoperative RT was significantly associated with improved OS compared to surgery alone (HR, 0.78; CI, 0.71 to 0.85; *p* < 0.001) (59).

Currently, there is no level I evidence for the benefits if RT in the management of RPS, and the results from retrospective analyses are also inconclusive (60). RT had no significant impact on distant metastasis or OS, thus making a selection of appropriate RT for managing PRPLS challenging (61).

Our study reviews current advances in the management of PRPLS in a single center. We showed that negative surgical margin is a prognostic factor of OS, which could prolong the postoperative survival time of PRPLS patients. Tumor stage is a prognostic factor for OS and PFS, and influences the survival of PRPLS patients, and earlier tumor stages are associated with significantly better outcomes.

## Data Availability Statement

The raw data supporting the conclusions of this article will be made available by the authors, without undue reservation.

## Ethics Statement

The studies involving human participants were reviewed and approved by the ethics committee of the Shanghai Jiaotong University Affiliated Sixth People's Hospital. Written informed consent for participation was not required for this study in accordance with the national legislation and the institutional requirements.

## Author Contributions

JC designed, coordinated the study, carried out the extraction of data, performed critical appraisal of the literature, and wrote the manuscript. YH assisted in review and collection of the clinical data and made statistical analysis. XH and QG supervised, assisted in the critical appraisal of included studies and critically reviewed the manuscript. YH contribute also majorly for paper revision. All authors contributed significantly to this work, read and approved the final manuscript.

## Conflict of Interest

The authors declare that the research was conducted in the absence of any commercial or financial relationships that could be construed as a potential conflict of interest.

## References

[1] Mack TM. Sarcomas and other malignancies of soft tissue, retroperitoneum, peritoneum, pleura, heart, mediastinum, and spleen. Cancer. (1995) 75:211–44. 10.1002/1097-0142(19950101)75:1+<211::AID-CNCR2820751309>3.0.CO;2-X 8000998

[2] Perez EA Gutierrez JC Moffat FL Franceschi D Livingstone AS Spector SA. Retroperitoneal and truncal sarcomas: prognosis depends upon type not location. Ann Surg Oncol. (2007) 14:1114–22. 10.1245/s10434-006-9255-x 17206483

[3] Singer S Antonescu CR Riedel E Brennan MF. Histologic subtype and margin of resection predict pattern of recurrence and survival for retroperitoneal liposarcoma. Ann Surg. (2003) 238:358–70. 10.1097/01.sla.0000086542.11899.38 14501502 PMC1422708

[4] van de Rijn M Fletcher JA. Genetics of soft tissue tumors. Annu Rev Pathol. (2006) 1:435–66. 10.1146/annurev.pathol.1.110304.100052 18039122

[5] Fletcher CDM Bridge JA Hogendoorn PCW Mertens F. WHO Classification of Tumours of Soft Tissue and Bone. Available online at: https://publications.iarc.fr/Book-And-Report-Series/Who-Classification-Of-Tumours/WHO-Classification-Of-Tumours-Of-Soft-Tissue-And-Bone-2013 (accessed April 3, 2021).

[6] Italiano A Bianchini L Keslair F Bonnafous S Cardot-Leccia N Coindre J-M. HMGA2 is the partner of mDM2 in well-differentiated and dedifferentiated liposarcomas whereas cDK4 belongs to a distinct inconsistent amplicon. Int J Cancer. (2008) 122:2233–41. 10.1002/ijc.23380 18214854

[7] Schwarzbach MHM Hohenberger P. Current concepts in the management of retroperitoneal soft tissue sarcoma. Recent Results Cancer Res. (2009) 179:301–19. 10.1007/978-3-540-77960-5_19 19230548

[8] Ferrario T Karakousis CP. Retroperitoneal sarcomas: grade and survival. Arch Surg. (2003) 138:248–51. 10.1001/archsurg.138.3.248 12611567

[9] Raut CP Pisters PWT. Retroperitoneal sarcomas: combined-modality treatment approaches. J Surg Oncol. (2006) 94:81–7. 10.1002/jso.20543 16788949

[10] Anaya DA Lahat G Liu J Xing Y Cormier JN Pisters PW. Multifocality in retroperitoneal sarcoma: a prognostic factor critical to surgical decision-making. Ann Surg. (2009) 249:137–42. 10.1097/SLA.0b013e3181928f2f 19106689

[11] Na JC Choi KH Yang SC Han WK. Surgical experience with retroperitoneal liposarcoma in a single Korean tertiary medical center. Korean J Urol. (2012) 53:310–6. 10.4111/kju.2012.53.5.310 22670189 PMC3364469

[12] Thomas JM. Retroperitoneal sarcoma. Br J Surg. (2007) 94:1057–8. 10.1002/bjs.5967 17701955

[13] Hughes TM Spillane AJ. Imaging of soft tissue tumours. Br J Surg. (2000) 87:259–60. 10.1046/j.1365-2168.2000.01412.x 10718790

[14] Tarone RE Ware J. On distribution-free tests for equality of survival distributions. Biometrika. (1977) 64:156–60. 10.1093/biomet/64.1.156

[15] Crago AM Singer S. Clinical and molecular approaches to well differentiated and dedifferentiated liposarcoma. Curr Opin Oncol. (2011) 23:373–8. 10.1097/CCO.0b013e32834796e6 21552124 PMC3253354

[16] Engström K Bergh P Gustafson P Hultborn R Johansson H Löfvenberg R. Liposarcoma: outcome based on the scandinavian sarcoma group register. Cancer. (2008) 113:1649–56. 10.1002/cncr.23784 18720363

[17] Bradley JC Caplan R. Giant retroperitoneal sarcoma: a case report and review of the management of retroperitoneal sarcomas. Am Surg. (2002) 68:52–6. 12467318

[18] Yol S Tavli S Tavli L Belviranli M Yosunkaya A. Retroperitoneal and scrotal giant liposarcoma: report of a case. Surg Today. (1998) 28:339–42. 10.1007/s005950050136 9548324

[19] Sioletic S Dal Cin P Fletcher CDM Hornick JL. Well-differentiated and dedifferentiated liposarcomas with prominent myxoid stroma: analysis of 56 cases. Histopathology. (2013) 62:287–93. 10.1111/j.1365-2559.2012.04348.x 23020289

[20] Mäkelä J Kiviniemi H Laitinen S. Prognostic factors predicting survival in the treatment of retroperitoneal sarcoma. Eur J Surg Oncol. (2000) 26:552–5. 10.1053/ejso.2000.0945 11034804

[21] Gladdy RA Qin L-X Moraco N Agaram NP Brennan MF Singer S. Predictors of survival and recurrence in primary leiomyosarcoma. Ann Surg Oncol. (2013) 20:1851–7. 10.1245/s10434-013-2876-y 23354568 PMC3657306

[22] Wu Y-X Liu J-Y Liu J-J Yan P Tang B Cui Y-H. A retrospective, single-center cohort study on 65 patients with primary retroperitoneal liposarcoma. Oncol Lett. (2018) 15:1799–810. 10.3892/ol.2017.7533 29434876 PMC5777090

[23] Zhao X Li P Huang X Chen L Liu N She Y. Prognostic factors predicting the postoperative survival period following treatment for primary retroperitoneal liposarcoma. Chin Med J. (2015) 128:85–90. 10.4103/0366-6999.147822 25563319 PMC4837826

[24] Nathenson MJ Barysauskas CM Nathenson RA Regine WF Hanna N Sausville E. Surgical resection for recurrent retroperitoneal leiomyosarcoma and liposarcoma. World J Surg Oncol. (2018) 16:203–8. 10.1186/s12957-018-1505-4 30309356 PMC6182828

[25] Ikoma N Torres KE Somaiah N Hunt KK Cormier JN Tseng W. Accuracy of preoperative percutaneous biopsy for the diagnosis of retroperitoneal liposarcoma subtypes. Ann Surg Oncol. (2015) 22:1068–72. 10.1245/s10434-014-4210-8 25354575 PMC4520392

[26] Gupta AK Cohan RH Francis IR Sondak VK Korobkin M. CT of recurrent retroperitoneal sarcomas. AJR Am J Roentgenol. (2000) 174:1025–30. 10.2214/ajr.174.4.1741025 10749244

[27] Chang I-YJ Herts BR. Retroperitoneal liposarcoma. J Urol. (2013) 189:1093–4. 10.1016/j.juro.2012.11.122 23201495

[28] Clark MA Thomas JM. Portsite recurrence after laparoscopy for staging of retroperitoneal sarcoma. Surg Laparosc Endosc Percutan Tech. (2003) 13:290–1. 10.1097/00129689-200308000-00015 12960797

[29] Fabre-Guillevin E Coindre J-M Somerhausen N de SA Bonichon F Stoeckle E Bui NB. Retroperitoneal liposarcomas: follow-up analysis of dedifferentiation after clinicopathologic reexamination of 86 liposarcomas and malignant fibrous histiocytomas. Cancer. (2006) 106:2725–33. 10.1002/cncr.21933 16688768

[30] Singer S Corson JM Demetri GD Healey EA Marcus K Eberlein TJ. Prognostic factors predictive of survival for truncal and retroperitoneal soft-tissue sarcoma. Ann Surg. (1995) 221:185–95. 10.1097/00000658-199502000-00009 7857146 PMC1234952

[31] Alvarenga JC Ball AB Fisher C Fryatt I Jones L Thomas JM. Limitations of surgery in the treatment of retroperitoneal sarcoma. Br J Surg. (1991) 78:912–6. 10.1002/bjs.1800780806 1913104

[32] Miao C Liu D Zhang F Wang Y Zhang Y Yu J. Association of FPGS genetic polymorphisms with primary retroperitoneal liposarcoma. Sci Rep. (2015) 5:9079. 10.1038/srep09079 25765001 PMC5390907

[33] Bonvalot S Miceli R Berselli M Causeret S Colombo C Mariani L. Aggressive surgery in retroperitoneal soft tissue sarcoma carried out at high-volume centers is safe and is associated with improved local control. Ann Surg Oncol. (2010) 17:1507–14. 10.1245/s10434-010-1057-5 20393803

[34] Erzen D Sencar M Novak J. Retroperitoneal sarcoma: 25 years of experience with aggressive surgical treatment at the institute of oncology, Ljubljana. J Surg Oncol. (2005) 91:1–9. 10.1002/jso.20265 15999353

[35] Joshi RM Gangurde GK Talathi NP Telavane PP Singh R Hanamshetti SR. Large retroperitoneal liposarcoma - a series of five cases. Indian J Surg. (2013) 75:64–8. 10.1007/s12262-011-0348-9 24426516 PMC3693309

[36] Bautista N Su W O'Connell TX. Retroperitoneal soft-tissue sarcomas: prognosis and treatment of primary and recurrent disease. Am Surg. (2000) 66:832–6. 10993610

[37] Park JO Qin L-X Prete FP Antonescu C Brennan MF Singer S. Predicting outcome by growth rate of locally recurrent retroperitoneal liposarcoma: the one centimeter per month rule. Ann Surg. (2009) 250:977–82. 10.1097/SLA.0b013e3181b2468b 19953716 PMC3248745

[38] Lewis JJ Leung D Woodruff JM Brennan MF. Retroperitoneal soft-tissue sarcoma: analysis of 500 patients treated and followed at a single institution. Ann Surg. (1998) 228:355–65. 10.1097/00000658-199809000-00008 9742918 PMC1191491

[39] Milone M Pezzullo LS Salvatore G Pezzullo MG Leongito M Esposito I. Management of high-grade retroperitoneal liposarcomas: personal experience. Updates Surg. (2011) 63:119–24. 10.1007/s13304-011-0061-z 21455814

[40] Wang Z Wu J Lv A Li C Li Z Zhao M. Infiltration characteristics and influencing factors of retroperitoneal liposarcoma: novel evidence for extended surgery and a tumor grading system. Biosci Trends. (2018) 12:185–92. 10.5582/bst.2018.01015 29657244

[41] Anaya DA Lahat G Wang X Xiao L Pisters PW Cormier JN. Postoperative nomogram for survival of patients with retroperitoneal sarcoma treated with curative intent. Ann Oncol. (2010) 21:397–402. 10.1093/annonc/mdp298 19622598

[42] Russo P Kim Y Ravindran S Huang W Brennan MF. Nephrectomy during operative management of retroperitoneal sarcoma. Ann Surg Oncol. (1997) 4:421–4. 10.1007/BF02305556 9259970

[43] Gronchi A Lo Vullo S Fiore M Mussi C Stacchiotti S Collini P. Aggressive surgical policies in a retrospectively reviewed single-institution case series of retroperitoneal soft tissue sarcoma patients. J Clin Oncol. (2009) 27:24–30. 10.1200/JCO.2008.17.8871 19047283

[44] Bonvalot S Rivoire M Castaing M Stoeckle E Le Cesne A Blay JY. Primary retroperitoneal sarcomas: a multivariate analysis of surgical factors associated with local control. J Clin Oncol. (2009) 27:31–7. 10.1200/JCO.2008.18.0802 19047280

[45] Tseng WW Wang SC Eichler CM Warren RS Nakakura EK. Complete and safe resection of challenging retroperitoneal tumors: anticipation of multi-organ and major vascular resection and use of adjunct procedures. World J Surg Oncol. (2011) 9:143. 10.1186/1477-7819-9-143 22054416 PMC3235074

[46] Mussi C Colombo P Bertuzzi A Coladonato M Bagnoli P Secondino S. Retroperitoneal sarcoma: is it time to change the surgical policy? Ann Surg Oncol. (2011) 18:2136–42. 10.1245/s10434-011-1742-z 21537866

[47] Linehan DC Lewis JJ Leung D Brennan MF. Influence of biologic factors and anatomic site in completely resected liposarcoma. J Clin Oncol. (2000) 18:1637–43. 10.1200/JCO.2000.18.8.1637 10764423

[48] Luo P Cai W Yang L Wu Z Chen Y Zhang R. Retroperitoneal dedifferentiated liposarcoma: analysis of 61 cases from a large institution. J Cancer. (2018) 9:3831–8. 10.7150/jca.25715 30410585 PMC6218781

[49] Fuks D Verhaeghe J-L Marchal F Guillemin F Beckendorf V Peiffert D. Surgery and postoperative radiation therapy in primary retroperitoneal sarcomas: experience of the cancer centre alexis-Vautrin. Cancer Radiother. (2012) 16:194–200. 10.1016/j.canrad.2011.11.006 22387193

[50] El-Bared N Taussky D Mehiri S Patocskai E Roberge D Donath D. Preoperative intensity modulated radiation therapy for retroperitoneal sarcoma. Technol Cancer Res Treat. (2014) 13:211–6. 10.7785/tcrt.2012.500371 23919397 PMC4527475

[51] Fabbroni C Fucà G Ligorio F Fumagalli E Barisella M Collini P. Impact of pathological stratification on the clinical outcomes of advanced well-differentiated/dedifferentiated liposarcoma treated with trabectedin. Cancers. (2021) 13:1453. 10.3390/cancers13061453 33810165 PMC8005098

[52] Tuan J Vitolo V Vischioni B Iannalfi A Fiore MR Fossati P. Radiation therapy for retroperitoneal sarcoma. Radiol Med. (2014) 119:790–802. 10.1007/s11547-013-0350-3 24638910

[53] Wortman JR Tirumani SH Tirumani H Shinagare AB Jagannathan JP Hornick JL. Neoadjuvant radiation in primary extremity liposarcoma: correlation of MRI features with histopathology. Eur Radiol. (2016) 26:1226–34. 10.1007/s00330-015-3953-3 26314480

[54] Pitson G Robinson P Wilke D Kandel RA White L Griffin AM. Radiation response: an additional unique signature of myxoid liposarcoma. Int J Radiat Oncol Biol Phys. (2004) 60:522–6. 10.1016/j.ijrobp.2004.03.009 15380587

[55] Engström K Bergh P Cederlund CG Hultborn R Willen H Åman P. Irradiation of myxoid/round cell liposarcoma induces volume reduction and lipoma-like morphology. Acta Oncol. (2007) 46:838–45. 10.1080/02841860601080415 17653909

[56] Roberge D Skamene T Nahal A Turcotte RE Powell T Freeman C. Radiological and pathological response following preoperative radiotherapy for soft-tissue sarcoma. Radiother Oncol. (2010) 97:404–7. 10.1016/j.radonc.2010.10.007 21040989

[57] Bonvalot S Gronchi A Le Péchoux C Swallow CJ Strauss D Meeus P. Preoperative radiotherapy plus surgery versus surgery alone for patients with primary retroperitoneal sarcoma (EORTC-62092: STRASS): a multicentre, open-label, randomised, phase 3 trial. Lancet Oncol. (2020) 21:1366–77. 10.1016/S1470-2045(20)30446-0 32941794

[58] Gronchi A Strauss DC Miceli R Bonvalot S Swallow CJ Hohenberger P. Variability in patterns of recurrence after resection of primary retroperitoneal sarcoma (RPS): a report on 1007 patients from the multi-institutional collaborative RPS working group. Ann Surg. (2016) 263:1002–9. 10.1097/SLA.0000000000001447 26727100

[59] Nussbaum DP Rushing CN Lane WO Cardona DM Kirsch DG Peterson BL. Preoperative or postoperative radiotherapy versus surgery alone for retroperitoneal sarcoma: a case-control, propensity score-matched analysis of a nationwide clinical oncology database. Lancet Oncol. (2016) 17:966–75. 10.1016/S1470-2045(16)30050-X 27210906

[60] Mullinax JE Zager JS Gonzalez RJ. Current diagnosis and management of retroperitoneal sarcoma. Cancer Control. (2011) 18:177–87. 10.1177/107327481101800305 21666580

[61] Haas RLM Bonvalot S Miceli R Strauss DC Swallow CJ Hohenberger P. Radiotherapy for retroperitoneal liposarcoma: a report from the transatlantic retroperitoneal sarcoma working group. Cancer. (2019) 125:1290–300. 10.1002/cncr.31927 30602058 PMC6590287

